# β-amyloid in biological samples: not all Aβ detection methods are created equal

**DOI:** 10.3389/fnagi.2014.00203

**Published:** 2014-08-13

**Authors:** Paul A. Adlard, Qiao-Xin Li, Catriona McLean, Colin L. Masters, Ashley I. Bush, Michelle Fodero-Tavoletti, Victor Villemagne, Kevin J. Barnham

**Affiliations:** ^1^The Florey Institute of Neuroscience and Mental HealthParkville, VIC, Australia; ^2^Department of Pathology, The University of MelbourneParkville, VIC, Australia; ^3^Bio21 Molecular Science and Biotechnology Institute, The University of MelbourneParkville, VIC, Australia

**Keywords:** amyloid, detection, ELISA, western blot, mass spectrometry

## Introduction

Alzheimer's disease (AD) is a progressive neurodegenerative disease and is the major cause of dementia in people aged 65 years and older, affecting approximately 2% of the population of industrialized countries (Mattson, 2004). The two primary histopathological lesions found within the AD brain are intracellular accumulations of hyper-phosphorylated tau protein, in the form of neurofibrillary tangles (NFTs), and extracellular deposits that are principally comprised of β-amyloid peptide (Aβ) (Adlard and Cummings, 2004). Aβ has been proposed to be a primary mediator of both the initiation and progression of disease (Hardy and Selkoe, 2002), with numerous toxic effects attributed to its abnormal accumulation within the neuropil (Karran et al., 2011). While there remains debate as to which precise species of Aβ represents the “toxic moiety” (Selkoe, 2008; Lublin and Gandy, 2010), the assessment of Aβ from bulk tissue remains one of the most common analyses conducted within the field, particularly as it relates to the development of therapeutic approaches to this intractable disease. Throughout our own investigations it became apparent that different commonly used methodologies for the quantitation of Aβ would often yield markedly different results. In this opinion piece we discuss this notion and present evidence that highlights the need to exercise caution when embarking on an analysis of Aβ, or indeed, when interpreting data from other papers. We also include some of our own data where we analyzed a common set of tissues using standard analysis techniques that are used in laboratories throughout the world, including western blot, ELISA and surface-enhanced laser desorption/ionization (SELDI) time of flight (TOF) mass spectrometry. These analyses are not meant as a strict side-by-side comparison of raw data generated by different methodologies, but rather, are designed to emulate different approaches that may be taken by different laboratories. Thus, whilst the antibodies, extraction buffers and other aspects of each technique do vary—this is the real-life situation where different approaches are taken to achieve the same endpoint analysis of Aβ burden. The resulting differences are striking, and whilst not necessarily unexpected—it is important that the field acknowledge this phenomenon and have an appreciation for the complexities involved in the apparently “simple” assessment of Aβ burden.

## Methods

The collection, processing, and storage of human brains for research purposes was conducted by the Victorian Brain Bank Network. Ethics approval was provided by The University of Melbourne Human Research Ethics Committee, application number 941478X, titled “Brain Banking for Neuroscience Research.”

### Tissue

A cohort of AD (*n* = 11 M, *n* = 3 F; 76 ± 2 years), FTD (*n* = 2 M, *n* = 3 F; 63 ± 6 years), DLB (*n* = 1 M, *n* = 4 F; 81 ± 5 years) and control (*n* = 7 M, *n* = 6 F; 73 ± 2 years) cases were obtained from the Victorian Brain Bank Network. Gray matter from the frontal and temporal cortices were sonicated in PBS and centrifuged at 100,000×g (30 min, 4°C). The protein content of the collected supernatant (soluble material) and pellet (insoluble material) fractions were assessed using the Pierce BCA protein assay kit according to manufacturers recommendations. The various fractions were subsequently analyzed using Western blot, ELISA and SELDI.

### Aβ assessment methods

For ELISA—Aβ levels were determined using the DELFIA® Double Capture ELISA as previously described (Ritchie et al., 2003). For western blots and SELDI-TOF mass spectrometry, Aβ levels were assessed as previously published (Adlard et al., 2008).

### Statistical analysis

**Figure 1:**
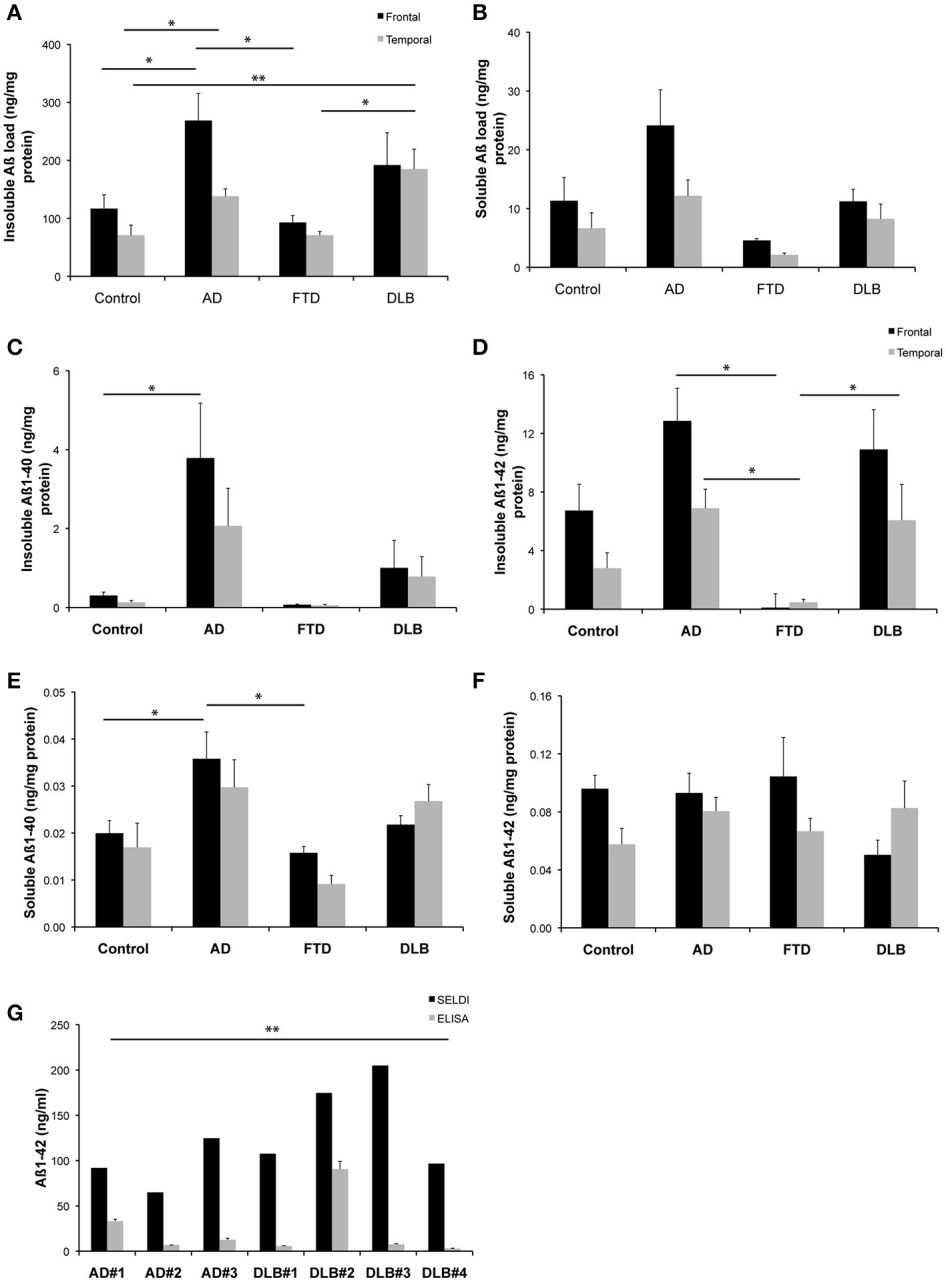
**Levels of Aβ detected in brain homogenates from a common set of human cases**. **(A,B)** Western blot assessment of soluble and insoluble Aβ burden across the entire cohort of neurological cases and healthy control cases utilized in this study **(C–F)** ELISA assessment of soluble and insoluble Aβ burden across the entire cohort of neurological cases and healthy control cases utilized in this study **(G)** A comparison of SELDI mass spectrometric and ELISA assessment of Aβ burden (Aβ 1-40 and Aβ 1-42) across a subset of the cases examined in this study. ^*^p < 0.05; ^**^p < 0.01.

A one-way analysis of variance with a Tukey's multiple comparison *post-hoc* test was utilized to analyse data in Figures 1A–F, while a paired *t*-test was utilized to analyse data in Figure 1G (GraphPad Prism 5.0 d).

## Results

The quantitation of the western blot data for both insoluble and soluble Aβ species across the different disease states is shown in Figures 1A,B. The highest levels of both insoluble and soluble Aβ species were present in the AD cases. This is consistent with the ELISA data for both insoluble and soluble Aβ species across the different disease states, as shown in Figures 1C–F. A statistical comparison of these data reveal that the ELISA assay reported significantly (*p* < 0.0002) lower levels of insoluble Aβ than the western blot assay (irrespective of whether the ELISA values for Aβ 1-40 and Aβ 1-42 are compared individually or summed for comparison to the western blot data). Likewise, the soluble data reveal the same difference between assay methodologies (*p* = 0.002).

Figure 1G shows a comparison of data generated from a subset of those samples used above utilizing either SELDI-TOF MS or ELISA techniques for the quantitation of Aβ across multiple AD and DLB cases. A pairwise comparison of the data generated by the respective methods for each individual case revealed that SELDI-TOF MS values for Aβ 1-42 were significantly higher (*p* < 0.05) than those obtained using ELISA.

## Discussion and opinion

In our own studies we had previously noted that common methodologies utilized for the assessment of protein levels in human biological fluids can return markedly different results when assessing levels of Aβ peptide. This highlighted the need for careful methodological considerations when embarking on our own analyses, or when interpreting the data reported from different laboratories. To formalize this, we assessed a common set of tissues using different techniques including western blot, ELISA and SELDI-TOF mass spectrometry, and demonstrated that, under the conditions utilized, both western blot and SELDI detected significantly larger pools of Aβ in human brain specimens than did ELISA assays. This was true for both soluble and insoluble fractions of human brain from a variety of different neurological disease states including AD, FTD, and DLB cases, as well as from age-matched controls. Whilst the antibodies, extraction buffers and other aspects of each technique did vary in this comparison—this is the real-life situation where different approaches are taken in different laboratories to achieve the same endpoint analysis of Aβ burden. A discussion of these methodological differences is provided below.

A similar phenomenon has previously been reported when examining tissues from cell culture media (HEK293 human embryonic kidney cells transfected with a plasmid vector containing the APP gene with either the Swedish or Arctic and Swedish mutations) and from transgenic animals (harboring the APP gene with either the Swedish or Arctic and Swedish mutations), where western blot quantitation revealed a markedly different Aβ profile than that shown by ELISA assay (Stenh et al., 2005). The authors determined that ELISA assays are inefficient at measuring Aβ oligomers and that different methodologies are required for the analysis of soluble Aβ. The data in this study are consistent with the over-arching finding of this previous report, and clearly demonstrate that Western blot and SELDI-TOF mass spectrometry are more sensitive techniques that detect a larger pool of Aβ in the human brain than does ELISA. Furthermore, western blot and SELDI offer the advantage that more information on the species of Aβ being quantitated are provided as a function of the methodology, where molecular weight is used to discriminate the various Aβ species present in a given sample. This represents perhaps one of the biggest advantages of these techniques over ELISA, which only provides an analysis of *total* immunoreactivity to a particular epitope within a sample, with no accounting for potential cross-reactivity with other proteins. This latter point is an important caveat, as data generated by ELISA, as with the other methodologies described, is dependent upon the specific antibodies utilized within the assay. There are a multitude of different antibodies that have been generated against different epitopes of the Aβ protein, and these may give different results when used in these assays. Likewise, as noted above, the specificity of the antibodies to a given target is critical, and is not always optimal when analysing biological samples. In the case of some commercial ELISA kits, the precise antibodies utilized (in addition to the extraction buffers that can liberate different pools of Aβ depending upon their composition) may also be proprietary, leaving the user essentially blind as to what species of Aβ they are specifically measuring. This may lead to a situation where the same samples measured using different ELISA kits provide very different quantitative results. This is a phenomenon that has been previously reported by Bjerke et al. (2010), who also noted that the source and quality of the Aβ utilized as a standard within the various ELISA kits often varies and may provide a source of variance that limits the cross-comparison of data generated using different ELISA kits. They also demonstrate that there are numerous confounding factors, other than the use of ELISA, in the analysis of Aβ from patient populations (the majority which result from the inherent properties of the Aβ protein itself).

The use of ELISA assays has become common-place in the AD field, representing the default approach to assay Aβ from a variety of biological fluids. The data presented in this paper, together with previous anecdotal and literature reports, however, suggest that the isolated use of ELISA for the quantitation of Aβ may be an insufficient and/or flawed approach. At a minimum, the reliance on ELISA data may result in an under-representation of the total Aβ protein levels present in a sample or, in the worse-case scenario, lead to a misinterpretation of data resulting from clinical trials where the assessment of efficacy of a given compound is the movement in a particular Aβ species, which may or may not be within the select “pool” of Aβ detected by an ELISA assay. This same caveat is also applicable to western blots and SELDI techniques, which are inherently reliant on discrete antibodies for their methods of detection. The difference, however, is that these latter techniques generally do not use proprietary antibodies and inherently provide a greater degree of detail on the precise Aβ species being examined. As the field continues to move forward, and our understanding of the relative pathogenicity of different Aβ species crystalizes, it is becoming apparent that the generic “bulk” assessment of Aβ burden is not sufficiently rigorous to provide the appropriate in-depth characterization, from both a basic science and a clinical perspective, that is required in patient populations. The development of new methodologies is critical, and more precise techniques such as mass spectrometry, which allows for the precise differentiation and quantitation of relevant analytes in a given sample, are now emerging as the preferred method for the critical analysis of Aβ from biological samples. Whilst these methods have their own limitations, in terms of cost, through-put and accessibility to the necessary infrastructure, their advantages are clear. Until such techniques become a more readily viable alternative for the field, the discussion and data presented in this paper highlight the need for a more rigorous approach to the assessment of Aβ that utilizes more common technologies such as western blot.

### Conflict of interest statement

The authors declare that the research was conducted in the absence of any commercial or financial relationships that could be construed as a potential conflict of interest.
